# Practical Notes and Formulæ

**Published:** 1883-12-20

**Authors:** 


### PRACTICAL NOTES AND FORMULÆ.

**Harness Polish.**—Useful to doctors.

Mutton suet........ .....................2 ounces,

Beeswax...............................6 "

Powdered sugar.......................4 "

Yellow soap...........................2 "

Lampblack............................1 "

Indigo.................................½ "

Water.................................¼ "

Oil of turpentine......................¼ "

Dissolve the soap in the water, add the other ingredients, except the turpentine, melt and mix well together. Finally, add the turpentine. The mixture is applied on the harness with a sponge, and polished with a brush.—*Drug Circular.*

**Creasote Wine,** recommended in phthisis, to diminish cough, fever, expectoration, etc., (New Remedies):

Wood creasote..............................6 parts,
Compound tinct. of gentian...............30 "
Alcohol.................................530 "
Sherry wine.............................710 "

M. Dose, a tablespoonful two or three times daily in a cupful of water.—*Lou. Med. News.*

**Iodoform in Hypertrophied Spleen.**—Dr. Francis A. Evans writes in Medical Summary: I have lately treated ten cases of hypertrophied spleen very successfully with the following formula:

℞ Iodoform..................................grs ij,
Saccharated ext. gelseminum..................grs. iv,
Digestin......................................grs. xij.

Triturate into twelve doses: giving two doses daily, morning and evening. Manipulation every evening by kneading. Oil the ague cake with simple cerate, or cerate and ext. hœdalia. The second course is sufficient. Give on seventh or eighth day a powder containing

℞ Podophyllin................................gr. ½,

Bitartrate potass..............................grs. iij, and the enlarged spleen is gone.

**Local Anæsthesia.**—The Medical News says local anæsthesia may be readily produced by applying, with a camel's hair brush, the following mixture:

℞ Chloral, } aa..............................℥ j,

Camphor, }

Morph. sulphat..............................ʒ ss,

Chloroform..................................ʒ j.

Sig. To be applied with a brush to the area to be incised.

3

**Application for the Removal of Scars.**—

℞ Borax.......................................℥ iss,

Salicylic acid.................................grs. xij,

Glycerine......................................ʒ iij,

Rose water.....................................℥ vj.

M.

Lint soaked in this solution and allowed to remain some little time each day will frequently mitigate the visible results of burns, small-pox scars, ringworm, etc.—*Med. Summary.*

**Eczema of the Head in Children.**—The following prescription has been highly recommended by Mr. Lassar in the form of eczema capitis observed in very young children:

℞. Acid salicylic..............................ʒ ss,

Tr. benzoin....................................ʒ j,

Ung. petroli...................................ʒ iij.

M. The child's head should first be thoroughly washed with castile soap, and then carefully anointed three times daily with the ointment.

If the crusts are hard and difficult to remove, they may be softened with olive oil containing two per cent. of salicylic acid.—*Med. and Surg. Rep.*

**Chronic Chills.**—We have found the following to be a very reliable remedy in chronic chills:

℞. Sulph. cinchondia.......................} aa. grs. xx,

Chenoidin..................................}

Podophylin.................................grs. iij,

Ipecac pulv................................grs. xv,

Capsicum pulv..............................grs. xx.

Make into five-grain pills.

M. Sig. One every three hours with water slightly acidulated with muriatic acid. We have not failed on a case using this.—*Atlanta Med. Jour.*

**Nausea and Vomiting in Uterine Affections.**—Dr. Cheron (in a foreign journal) found great benefit result from the administration of bromides in an effervescing mixture, of which the following is the formula:

No. 1.—℞. Potass. bicarb.......................gr. xxx,
Potass. bromid.................................gr. xxx,
M. Aquæ........................................℥ ii.

No. 2.—℞. Acid. citric.........................gr. lx,

Syrup simp.....................................℥ i,

M. Aquæ........................................℥ iv.

A teaspoonful of No. 1 to be mixed with a tablespoonful of No. 2, and the mixture to be taken during effervescence. The dose may be repeated every hour or half-hour—the quantities stated in the above formula representing the maximum to be taken per diem.

